# Differences in the Properties and Mirna Expression Profiles between Side Populations from Hepatic Cancer Cells and Normal Liver Cells

**DOI:** 10.1371/journal.pone.0023311

**Published:** 2011-08-03

**Authors:** Wei-hui Liu, Kai-shan Tao, Nan You, Zheng-cai Liu, Hong-tao Zhang, Ke-feng Dou

**Affiliations:** 1 PLA Center of General Surgery, General Hospital of Chengdu Army Region, Chengdu, Sichuan Province, China; 2 Department of Hepatobiliary Surgery, Xijing Hospital, Fourth Military Medical University, Xi'an, Shaanxi Province, China; National Cancer Institute, United States of America

## Abstract

**Aims:**

Because hepatic cancer stem cells (HCSCs) are believed to derive from the conversion of hepatic normal stem cells (HNSCs), the identification of the differences that distinguish HCSCs from HNSCs is important.

**Methods:**

The HCC model was established in F344 rats by DEN induction. Using FACS analysis, side population cells from HCC (SP-HCCs) were isolated from the epithelial-like cells of HCC tissues, and the side population cells from normal liver (SP-NLCs) were isolated from syngeneic normal liver cells. The expression of stem cell markers was detected in both freshly isolated and amplified subpopulations. After induction with HGF, the differentiation of each subpopulation was analyzed by detection of early and late liver markers. In vivo, the biological characteristics of SP-HCCs and SP-NLCs were analyzed by repairing injured livers or forming tumors in nude mice. In addition, the expression of miRNAs was examined in both populations by miRNA array and QRT-PCR.

**Results:**

SP-NLCs and SP-HCCs were 4.30±0.011% and 2.100±0.010% of the whole population, respectively. Both SP-NLCs and SP-HCCs displayed greater expression of stem cell markers (CD133 and EpCAM) than NSP-NLCs and NSP-HCCs, respectively (*P*<0.01), both after fresh isolation and amplification. Upon HGF induction, SP-NLCs generated many ALB positive cells and few CK-7 positive cells, but NSP-NLCs could generate only ALB positive cells. In contrast, SP-HCCs gave rise to only AFP positive cells. As few as 5×10^5^ SP-NLCs were capable of repairing liver injury, while the same number of NSP-NLCs could not repair the liver. Furthermore, only 1×10^4^ SP-HCCs were necessary to initiate a tumor, while NSP-HCCs could not form a tumor. Compared to SP-NLCs, 68 up-regulated and 10 down-regulated miRNAs were present in SP-HCCs (*P*<0.01).

**Conclusion:**

Based on the decisive roles of some miRNAs in the genesis of HCSCs, miRNAs may contribute to the different characteristics that distinguish SP-HCCs from SP-NLCs.

## Introduction

Increasing evidence has shown that cancers contain a small subset of their own stem-like cells, called “cancer stem cells” (CSCs) [1], [2], [3], which are mostly affected by both tumor suppressors and cancer inducers [4], [5], [6]. HCC also contains hepatic CSCs (HCSCs), which have the greatest potential to proliferate and invade surrounding tissue [7]. Recent publications have shown that HCSCs may originate from hepatic normal stem cells (HNSCs) [8]. Even the initial event that transforms HNSCs to HCSCs is proposed to be a form of deregulation of HNSCs self-renewal [9]. Thus, comparing the characteristics of HNSCs and HCSCs is important. Normal liver is rich in HNSCs [10], and the suggestion that these HNSCs may serve as an optimal control for studying the characteristics of HCSCs is reasonable. However, molecular markers that define both HCSCs and HNSCs remain controversial; therefore, the isolation of side population (SP) cells has been widely used to enrich both types of stem cells [11]. SP cells have been demonstrated to be immature and undifferentiated cells and to express high levels of some specific stem cell markers [12]. Hence, the isolation of SP cells is an alternative source of stem cells, which is particularly useful in situations in which stem cell markers are unknown [12]. In mice and rats, the SP phenotype appears to be a common feature of stem cells, including normal and cancer stem cells [13], [14], [15]. In the liver, these SP cells have been shown to serve a central role in liver regeneration and liver cancer [16]. Therefore, SP cells can be considered appropriate alternatives to study HNSCs and HCSCs.

MiRNAs are emerging as important regulators of post-transcriptional gene regulation. The importance of miRNAs is underscored by the fact that they are often deregulated during carcinogenesis [17], [18], [19], [20]. Some miRNAs can promote tumor growth through common mechanisms that contribute to miRNA-regulated cell cycle control [21]. In addition, miRNAs have been demonstrated to be an integral component of stem cell regulation, including normal stem cells (NSCs) and CSCs [22]. A perturbation of key miRNA-mRNA networks in NSCs has been suggested to be a hallmark of CSCs [23]. In fact, a single oncogene (miRNA-145) has been demonstrated to re-program primary cells to display a CSCs phenotype [24]. Thus, the identification of common and unique expression patterns of miRNAs between HCSCs and HNSCs is essential.

In this study, we applied SP analysis to two different populations of primary cultured epithelial cells. One cell type was isolated from rat HCC tissues induced by diethylinitrosamine (DEN) and the other cell type was isolated from syngeneic rat liver tissues. Side populations from normal liver cells (SP-NLCs) and from HCCs (SP-HCCs) highly expressed stem cell markers. *In vitro*, both SP cells had high capacities to proliferate and could differentiate into mature cells upon induction with hepatocyte growth factor (HGF). *In vivo*, SP-NLCs could greatly aid in repairing an injured rat liver. In contrast, SP-HCCs could initiate tumors both in subcutaneous and liver tissues of Non-obese diabetic/severe combined immunodeficiency (NOD/SCID) mice. Assuming that these differences were related to the vastly different expression patterns of miRNAs between these two cell populations, we examined the miRNA profiles of SP-NLCs and SP-HCCs. Because HCSCs are proposed to be HCC initiating cells, identifying the differences between SP-HCCs and SP-NLCs, including deregulated miRNAs, may greatly aid in understanding the genesis of HCSCs and the tumorigenesis of HCC.

## Materials and Methods

### 1. Specimen collection

Thirty male Fisher 344 rats (from the National Rodent Laboratory Animal Resource, Shanghai, China) were randomly divided into control and trial groups. Rats in the trial group were treated with 0.05% DEN (Sigma Co, USA) in their drinking water for 6 weeks and were then changed to normal drinking water [25], whereas rats in the control group were given a normal diet. Three rats from each group were sacrificed under anesthesia at 2, 6, 10, 14 and 18 weeks after DEN induction. Both HCC nodules from the trial group and normal livers from the control group were collected. Portions of these tissues were fixed in 10% phosphate-buffered neutral formalin and routinely processed and stained with Hematoxylin and Eosin (H&E) for histological examination. The remaining tissues were used directly in the experiments detailed below. All animal experiments were performed in accordance with animal study protocols [26] and approved by the Research Animal Care and Use Committee at the Fourth Military Medical University. The animal protocol number was SYXK2008-005.

### 2. Cell isolation and culture

Hepatic cancer cells (HCCs) were isolated according to Hohne et al. [27] with minor modifications, and normal liver cells (NLCs) were isolated according to Oertel et al. [28]. Both HCC and normal liver (NL) tissues were minced in Dulbecco's modified eagle's medium (DMEM) (Invitrogen Co, USA) with 0.1% collagenase type IV and 0.005% trypsin (Sigma Co, USA) and then incubated for 20 min at 37°C in a shaking water bath. After incubation, supernatants containing the released cells were passed through a 100 µm nylon mesh and centrifuged at 1,000× g for 8 min. The pellets were washed twice with phosphate-balanced saline (PBS) (Invitrogen Co, USA), and single cell suspensions were collected. The NL single cell suspension was centrifuged for 5 min at 100× g in DMEM, and the supernatant was collected. A Percoll (Invitrogen Co, USA) gradient was prepared in a 50 ml tube by sequentially layering 10 ml of 70%, 50% and 30% Percoll. A total of 20 ml of NLCs in PBS was added, and the tube was centrifuged at 1000× g for 10 min. The cell fraction at the interface between 30% and 50% Percoll was collected. Both NLCs and HCCs were cultured in 6-well plates containing William's E Medium (Sigma Co, USA) supplemented with 10% vol/vol fetal bovine serum (Invitrogen Co, USA), 5 µg/ml insulin (Sigma Co, USA), 5 µM hydrocortisone, 100 U/ml penicillin and 100 µg/ml streptomycin at 37°C in a humidified atmosphere of 5% CO_2_. Adherent cells proliferated and extended as a monolayer colony after 20 days in culture. We collected the monoclonal cell population by local digestion with cloning cylinders and transferred the cells into a new culture dish to continue the culturing process.

### 3. SP Cell sorting

The cells were divided into two portions: half was directly used as a sham sorted population (SSP), while the other half was used for cell sorting on a FACS Vantage II cell sorter (Becton Dickinson Co, USA). The following information describes our isolation protocol. Cells were labeled with Hoechst 33342 dye (Sigma Co, USA) at a final concentration of 4 mg/ml in the presence or absence of 50 µM verapamil (Sigma Co, USA) and incubated at 37°C for 90 min according to the methods described by Goodell et al. [11]. The stained cells were washed with ice-cold PBS containing 2% bovine serum albumin (BSA) and 10 mM HEPES, centrifuged at 4°C and resuspended in the same buffer. Propidium iodide (PI) (Sigma Co, USA) was used to detect cell viability. Hoechst 33342 was excited at 355 nm and its fluorescence was analyzed at two wavelengths: Hoechst 33342 blue at 450 nm and Hoechst 33342 red at 675 nm. A second 488 nm argon laser (100 mW) was used to excite PI fluorescence for excluding dead cells. SP cells showed low staining with Hoechst and non-side population (NSP) cells were more brightly stained.

### 4. Cell growth test

This experiment was employed to evaluate the proliferative ability of the cells from each subpopulation, including SP, NSP and SSP. The cells in each subpopulation were adjusted to 2×10^6^/ml and seeded in 32 flasks (0.5×10^5^ cells per flask). The culture media was supplemented with leukemia inhibitory factor (LIF) at a concentration of 10 µg/ml. Every day during a period of 7 days, 4 parallel cell samples from each subpopulation were trypsinized and counted under an inverted microscope (BX50-32E01, Olympus, Tokyo, Japan).

### 5. Detection of stem cell markers by fluorescent activated cells sorting (FACS)

The expression of stem cell markers was analyzed by a FACSCaliburTM system (BD Immunocytometry Systems, San Jose, CA) in both freshly isolated subpopulations and amplified subpopulations. Briefly, the cells were incubated in William's E Medium (containing 20% FBS) at 10^6^ cells/ml for 15–30 min at room temperature to block non-specific sites for antibody binding. The cells from different subpopulations were washed twice with PBS and re-suspended in 990 µl PBS. Subsequently, 10 µl of antibodies, including CD133 (PE conjugated, Biolegend, USA) and EpCAM (fluorescein isothiocyanate (FITC) conjugated, Biolegend, USA), were added to each cell suspension. After 30 min of incubation at 4°C in the dark, the cells were washed twice with PBS, fixed in 0.1% formaldehyde and analyzed by flow cytometry.

### 6. Cell induction by HGF

The cells from each subpopulation were cultured in induction media, which was commercial serum-free medium (Sigma Co, USA) supplemented with HGF (20 ng/ml). The cell differentiation was evaluated by detecting the expression of liver-specific markers as described below.

### 7. Detection of liver markers by immunofluorescence (IF)

After induction by HGF, IF was performed to qualitatively evaluate whether the induced cells expressed specific liver markers. To identify bi-directional differentiation of the different populations in NLCs (SP-NLCs, NSP-NLCs and SSP-NLCs), two specific primary markers were selected: the mature hepatic marker albumin (ALB) (dilution 1∶200; Santa Cruz, CA) and the biliary marker cytokine 7 (CK-7) (dilution 1∶200; Santa Cruz, CA). To identify maturation of different populations in HCCs (SP-HCCs, NSP-HCCs and SSP-HCCs), the tumor markers alpha fetoprotein (AFP) (dilution 1∶200; Santa Cruz, CA) and CK-19 (dilution 1∶200; Santa Cruz, CA) were selected. Briefly, with the culture medium removed, cells on the culture slide were rinsed twice with PBS, fixed with 4% paraformaldehyde for 20 min and then immersed in PBS for 10 min, followed by exposure to 0.01% Triton X-100 at room temperature for 10 min. For blocking non-specific immune reactions, the cells were treated with 6% goat serum (Santa Cruz, CA) at room temperature for 30 min. The cells cultured in each slide were subjected to primary antibodies at 4°C overnight and were washed three times with cold PBS. The fluorescent FITC-conjugated goat anti-rabbit secondary antibody (dilution 1∶100; Santa Cruz, CA) was added and incubated for 2 h. Subsequently, the cells were treated with 2-(4-Amidinophenyl)-6-indolecarbamidine dihydrochloride (DAPI) (dilution 1∶100; Sigma) for 15 min. The fluorescence was observed through an appropriate filter using a fluorescence microscope (FV1000MPE, Olympus Co, Tokyo, Japan).

### 8. Detection of liver markers by western blotting

After induction by HGF, western blotting was performed to quantitatively detect specific liver markers in the induced cells. ALB and CK-7 were examined in SP-NLCs, NSP-NLCs and SSP-NLCs; AFP and CK-19 were analyzed in SP-HCCs, NSP-HCCs and SSP-HCCs. Cells were lysed in whole-cell extraction buffer (RIPA buffer) containing a protease inhibitor cocktail tablet (Complete-Mini, Roche Diagnostics, Mannheim, Germany). The homogenates were centrifuged at 3000× g for 20 min at 4°C, and the supernatants were collected. Proteins were separated on 12% SDS-polyacrylamide gel and transferred to an Immobilon-P PVDF (polyvinylidene fluoride) membrane (MILLIPORE, Billerica, MA, USA). The blots were saturated with blocking buffer (5% skim milk in TBS-T) for 1 h at room temperature and then incubated overnight at 4°C with rabbit anti-human/rat/mouse monoclonal antibodies (1∶600; Santa Cruz Biotechnology, Inc., Santa Cruz, CA) and a glyceraldehyde-3-phosphate dehydrogenase (GAPDH) antibody (1∶600; Sigma, Saint Louis, MO). After washed in TBS-T, the membranes were incubated for 1 h at room temperature with HRP-Goat Anti-Rabbit IgG (1∶2000; Perkin Elmer, Inc., Waltham, MA). Detection of the proteins was performed using an ECL system (Cell Signaling Technology, Beverly, MA, USA). The grayscale values of each band on the blots were measured using BandScan4.3.

### 9. Liver injury model and cell transplantation

SP-NLCs and NSP-NLCs were independently washed with PBS in the dark and resuspended in 2 ml staining solution to label the cell membrane with red fluorescence at 37°C, according to the protocol supplied with the PKH26 red fluorescent cell linker kit (Sigma Corp., USA). Serum-containing media was added to the staining solution to terminate the staining 5 min later. Stained cells were washed three times with PBS and suspended in 0.5 ml PBS for transplantation. To induce liver injury, 20 normal F344 rats (10 for SP-NLCs transplantation, 10 for NSP-NLCs injection) were administered CCl_4_ intraperitoneally at a dose of 1.2 ml/kg body weight and received a two-thirds partial hepatectomy (2/3 PH) three days later. Immediately after PH, the prepared cells (5×10^5^ cells per rat) were separately injected into these rats through the portal vein.

For each liver, we randomly cut four frozen sections. To evaluate the colonization effects of SP-NLCs and NSP-NLCs, the restored liver sections were viewed under an inverted microscope. When red areas were observed in the sections, the result was identified as positive. Under each field of view, the positive areas were counted, and the percentage of the positive area relative to the whole area was calculated. A percent of red area of <5% was defined as negative (−), 5–25% as positive (+), 25–50% as moderately positive (++) and >50% as strongly positive (+++).

### 10. NOD/SCID xenograft transplant experiments

Different numbers (1×10^7^, 1×10^6^, 1×10^5^ and 1×10^4^) of SP-HCCs or NSP-HCCs were injected into NOD/SCID mice by subcutaneous injection. Each group contained 4 mice; thus, 32 mice were used for xenotransplantation. Each mouce were done with 4 injections, symmetrically 2 injections in left back and 2 injections in right back. Tumor growth was monitored every 2 days after the second week of inoculation. All mice were sacrificed at day 60. All of the tumor tissues were collected, fixed in 4% formaldehyde, and embedded in paraffin for H&E staining to assess tumor histology. All the results were judged by three different researchers independently. We summarized the data and calculated the average diameter of tumors in each group (such as 1×10^7^ SP-HCCs group). According to the average size of tumors, they were divided into four different grades: grade 1 (−), no macroscopic tumor; grade 2 (+), the diameter of tumor <0.2 cm; grade 3 (++), 0.2–0.5 cm; grade 4 (+++), >0.5 cm.

### 11. The expression of miRNAs in SP cells

Total RNAs were obtained from both SP-NLCs and SP-HCCs by the Totally RNA isolation kit (Ambion, Austin, TX). The quality and quantity of total RNAs were checked by 1.5% agarose gel electrophoresis and ultraviolet quantitation. The expression profiles of miRNAs were then detected by the miRCURY LNA™ (locked nucleic acid) microRNA Arrays Kit (Exiqon Co, Denmark), which covers all human, mouse and rat miRNA antisense sequences. In addition, the kit also incorporated 144 miRPlus™ probes, which were provided by Exiqon Corporation for novel miRNA detection. One microgram of RNA from SP-HCCs, SP-NLCs and reference pools were co-hybridized onto the Exiqon miRNA Array for 16 hr at 56°C. After incubation with Cy3-labeled dendrimers (Genisphere Inc, Hatfield, PA) [29], the microarrays were washed consecutively with wash buffers A, B and C. The fluorescent signals on the hybridized array were captured by a GenePix 4000B scanner and quantified using GenePix Pro4.0 (Axon Instruments, Burlingame, CA). Data manipulation was facilitated with Normalization Suite v1.63 (Ontario Cancer Institute, Toronto, Canada) [30]. The test to reference ratio for each miRNA was averaged from triplicate spots and between replicate experiments. Ratios greater or less than two-fold were considered to be up-regulated or down-regulated, respectively.

Two highly up-regulated miRNAs, three slightly up-regulated miRNAs, one greatly down-regulated miRNA and one moderately down-regulated miRNA were selected as representative miRNAs to be validated by quantitative real time polymerase chain reaction (QRT-PCR). Total RNAs were reverse-transcribed by MultiScribe (Applied Biosystems) in reaction mixtures containing miR-specific stem-loop reverse-transcription (RT) primers (Table 1). The PCR primers are listed in Table 1, and the cycle parameters for the PCR reaction were 95°C for 15 min followed by 40 cycles of a denaturation step at 95°C for 15 sec and an annealing/extension step at 60°C for 60 sec. All reactions were run in triplicate. The relative amount of each miRNA to U6 RNA was described by the equation ΔC_T_ = (C_T_miRNA−C_T_U6) [31]. The fold change in miRNAs from SP-HCCs compared with SP-NLCs are shown using the equation 2^−ΔΔCT^, where ΔΔC_T_ = (ΔC_T_ SP-HCCs−ΔC_T_ SP-NLCs).

**Table 1:** Oligonucleotides used in the QRT- PCR.

Name	RT primer (5′-3′)	PCR Forward primer (5′-3′)	*Tm* (°C)
U6	**CGCTTCACGAATTTGCGTGTCAT**	GCTTCGGCAGCACATATACTAAAAT	60
has-miR-10b	GTCGTATCCAGTGCAGGGTCCGAGGTATTCGCACTGGATACGAC**CACAAA**	**CATGG**TACCCTGTAGAACCGAA	60
has-miR-21	GTCGTATCCAGTGCAGGGTCCGAGGTATTCGCACTGGATACGAC**TCAACA**	**CGCGC**TAGCTTATCAGACTGA	60
hsa-miR-34c-3p	GTCGTATCCAGTGCAGGGTCCGAGGTATTCGCACTGGATACGAC**CCTGGC**	**GGTGG**AATCACTAACCACACG	60
hsa-miR-16	GTCGTATCCAGTGCAGGGTCCGAGGTATTCGCACTGGATACGAC**CGCCAA**	**CGCGC**TAGCAGCACGTAAATA	60
has-let7i*	GTCGTATCCAGTGCAGGGTCCGAGGTATTCGCACTGGATACGAC**AGCAAG**	**TAGTA**CTGCGCAAGCTACTGC	60
has-miR-200a*	GTCGTATCCAGTGCAGGGTCCGAGGTATTCGCACTGGATACGAC**TCCAGC**	**GAGTG**CATCTTACCGGACAGT	60
has-miR-148b*	GTCGTATCCAGTGCAGGGTCCGAGGTATTCGCACTGGATACGAC**GCCTGA**	**GGCGC**AAGTTCTGTTATACAC	60
General primer	PCR Reverse Primer: **GTGCAGGGTCCGAGGT**		60

### 12. Targets of deregulated miRNAs

#### 12.1. Prediction of potential targets for deregulated miRNAs

The potential targets for the deregulated miRNAs found by the above methods were predicted by two publicly available algorithms, including MiRBase Targets version 5 (available at: http://microrna.sanger.ac.uk/) and Targetscan version 4.2 (http://www.targetscan.org/).

#### 12.2 Identification of targets by semi-quantitative real time polymerase chain reaction (sQRT-PCR)

We summarized the proven targets of seven validated deregulated miRNAs. Among these targets, the miR-200a* target genes ZEB1 and ZEB2 [32], [33] were analyzed in both SP-HCCs and SP-NLCs by sQRT-PCR. Total RNA was extracted from cells using Trizol reagent (Molecular Research Center, Cincinnati, OH), and reverse transcribed into cDNA by SuperScript II Reverse Transcriptase according to the manufacturer's instructions (Invitrogen, Carlsbad, CA). Equal amounts of cDNA from these two samples were amplified with the following specific primers: ZEB1 (Sense 5′- AAGAAAGTGTTACAGATGCAGCTG-3′, Antisense 5′- CCCTGGTAACACTGTCTGGTC-3′); and ZEB2 (Sense 5′-ATACCAGCGGAAACAAGGATTTCA-3′, Antisense 5′-CAGGAATCGGAGTCTGTCAAGTCA-3′). The number of PCR cycles was 35. Each cycle consisted of denaturation step at 95°C for 30 s, primer annealing step at 65°C for 30 s and extension step at 72°C for 45 s. The PCR products were analyzed by 1.5% agarose gel electrophoresis stained with ethidium bromide.

### 13. Statistical analyses

Data are expressed as the mean ± standard error from at least three separate experiments performed in triplicate. Differences between groups were analyzed with SAM software version 3.0 using a double-sided Student's *t*-test when only two groups were present, and the null hypothesis was rejected at the 0.05 level.

## Results

### 1. Tissues preparation and cell culture

**Figure 1:**
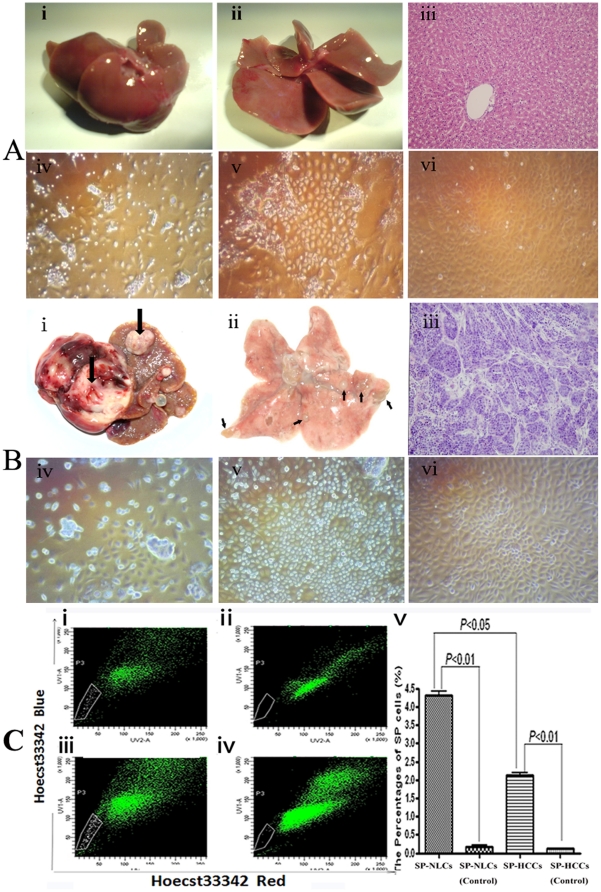
Upper panel: normal liver segregation and cell culture. (A-i) Morphology of livers in the non-DEN treated group, (A-ii) Thin-sliced sections reveal completely normal liver tissue, (A-iii) Histological features of normal liver with a regular structure. Primary cultured NLCs for (A-iv) 4 days, (A-v) 15 days and (A-vi) 25 days. Middle panel: primary HCC tissue segregation and cell culture. (B-i) Multiple primary HCC nodules in the rat liver, one of which is indicated by an arrow, (B-ii) Metastatic HCC nodules in rat lungs, which are indicated by arrows, (B-iii) Histological features of metastatic HCC tissue, in which normal lung lobules were replaced by carcinoma masses; Primary cultured HCC cells for (B-iv) 4 days, (B-v) 15 days and (B-vi) 30 days. Lower panel: the isolation of different subpopulations. (C-i) Without verapamil: SP cells were shown as a percentage of the NLCs; (C-ii) With verapamil: the profile of SP cells decreased greatly. (C-iii) Without verapamil: SP cells were shown with low fluorescence in HCCs; (C-iv) With verapamil: fluorescence of the SP cells fraction shifted to a higher level. (C-v) The percentages of SP cells in different groups are reflected in a column chart. Original magnification, 200× (A, B-iii), 100× (A, B-iv, v, vi).

NL tissues obtained from the control group were bright red and displayed smooth surfaces (Figure 1A-i). When these livers were cut into thin sections, completely normal liver tissue was revealed (Figure 1A-ii). Upon H&E staining, the liver lobules were observed to be in good order (Figure 1A-iii). Small NLCs were selected by Percoll discontinuous gradient centrifugation (PDGC) and cultured. NLCs formed clones after approximately 8 days of culture (Figure 1A-iv). After culturing for 15 days, these small cells covered approximately 65% of the plate (Figure 1A-v). After 25 days, these homogeneous cells almost fully covered the plates (Figure 1A-vi).

Small tumors were first found in rats sacrificed 8 weeks after DEN induction. After another 10 weeks, two-thirds of the livers contained tumor tissues with rough surfaces (Figure 1B-i). We also found numerous metastatic cancer nodules in the lungs (Figure 1B-ii). Three different pathologists assessed the H&E staining and verified that these neoplasms were all of hepatic origin (Figure 1B-iii). The cells isolated from the primary HCC tissues grew slowly at first with only a few clones formed (Figure 1B-iv). After 15 days, the cells proliferated rapidly and covered 60% of each plate (Figure 1B-v). One month later, these cells fully covered the plates (Figure 1B-vi).

### 2. Isolation of SP cells by FACS

In the NLCs group, the percentage of SP cells was 4.300%±0.011% (Figure 1C-i). When exclusion of the dye was inhibited by verapamil in the control group, SP cells were nearly identical to the rest of the cells (Figure 1C-ii). The percentage of SP cells in the HCCs group was 2.100%±0.010% (Figure 1C-iii). When the exclusion of the dye was inhibited by verapamil, these SP cells also could not be discriminated from their controls (Figure 1C-iv). The profile of SP cells in NLCs was significantly higher than that in HCCs (*P*<0.05) (Figure 1C-v).

### 3. Self-renewal of SP cells

**Figure 2:**
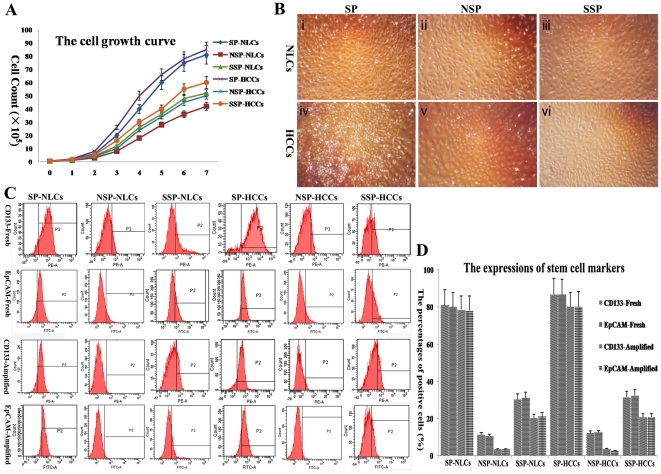
The self-renewal analysis of each subpopulation. (A) The cell growth curve during 7 days culture. (B) After amplification, distinct cell densities were observed in the different subpopulations. (C) The expression of stem cell markers (CD133 and EpCAM) was different in each freshly isolated and amplified subpopulation by FACS. (D) The exact data were reflected by a column chart. CD133/EpCAM-Fresh indicates the expression of CD133/EpCAM in freshly isolated subpopulations, and CD133/EpCAM-Amplified means the expression of CD133/EpCAM in amplified subpopulations. Original magnification, 100× (B).

Two standard features are characteristics of stem cells: self-renewal and multipotency. For self-renewal, SP-HCCs proliferated the fastest during 7 days culture, followed by SP-NLCs, SSP-HCCs, SSP-NLCs and NSP-HCCs (which proliferated similarly), and, finally, the NSP-NLCs (Figure 2A). Generally speaking, SP cells proliferated much faster than both NSP cells and SSP cells (*P*<0.01), and HCCs proliferated a little faster than NLCs. Because the initial number of each cell population was the same, totally different cell numbers were present in each group at the end of the culture period (Figure 2B). SP cells (Figure 2B-i,iv) were found to be more homogeneous and much smaller in size than both NSP cells (Figure 2B-ii,v) and SSP cells (Figure 2B-iii,vi) (*P*<0.01). However, no significant morphological differences were observed between SP-NLCs (Figure 2B-i) and SP-HCCs (Figure 2B-iv). Thus, these two populations could not be discriminated from each other under an inverted microscope.

At the very beginning of culture, both SP-NLCs and SP-HCCs expressed more of the stem cell markers than NSP-NLCs and NSP-HCCs, respectively (*P*<0.01) (Figure 2C). The following percentages of positive cells in each subpopulation were observed: CD133 percentages in SP-NLCs, NSP-NLCs, SSP-NLCs, SP-HCCs, NSP-HCCs and SSP-HCCs were 81.2±7.08, 11.4±1.31, 30.3±3.21, 86.7±8.32, 12.7±1.39 and 31.6±3.42, respectively; EpCAM percentages in these subpopulations were 80.1±8.10, 10.6±1.21, 31.2±3.18, 86.5±3.28, 12.4±1.31 and 32.6±3.67, respectively. At the end of the culture period, SP-NLCs and SP-HCCs still expressed more of the stem cell markers than NSP-NLCs and NSP-HCCs, respectively (*P*<0.01) (Figure 2D). Furthermore, the expression of stem cell markers decreased much more slowly in SP cells than in both SSP cells and NSP cells (*P*<0.01). The following percentages were observed: CD133 percentages in SP-NLCs, NSP-NLCs, SSP-NLCs, SP-HCCs, NSP-HCCs and SSP-HCCs were 78.6±6.98, 3.4±0.33, 20.2±2.03, 80.5±7.86, 3.6±0.30 and 20.7±2.38, respectively; EpCAM percentages in these subpopulations were 78.1±7.53, 3.5±0.28, 21.5±2.17, 80.3±8.12, 2.3±0.27 and 20.2±2.28, respectively. During the proliferation period, both SP-NLCs and SP-HCCs maintained the high expression of stem cell markers; in contrast, both NSP-NLCs and NSP-HCCs gradually lost expression of the stem cell markers. These data suggest that SP cells are similar to stem cells in their self-renewal capacity.

### 4. Differentiation of SP cells induced by HGF in vitro

**Figure 3:**
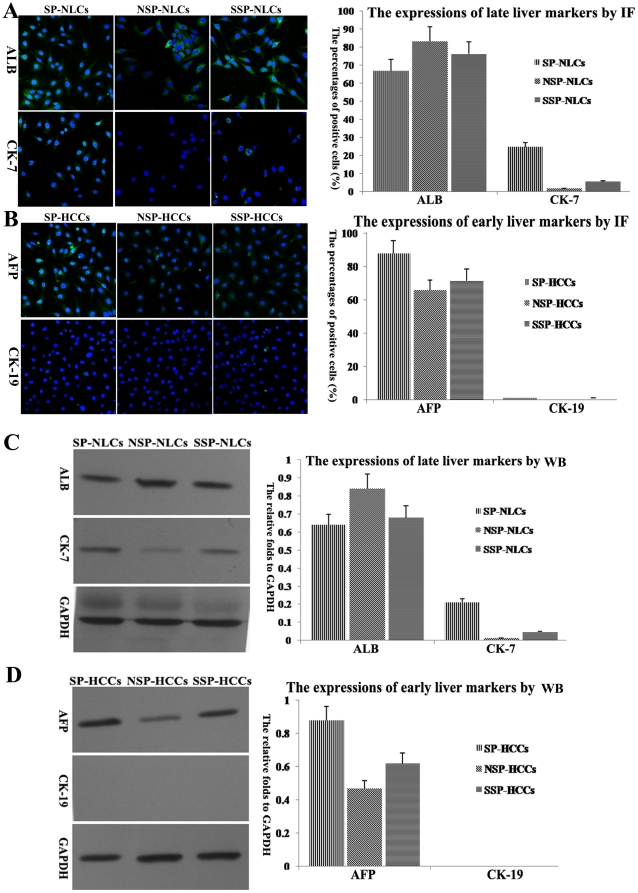
The induced differentiation of each subpopulation. (A) Through IF, ALB positive cells (green, nuclei in blue) and CK-7 positive cells (green, nuclei in blue) were differentially produced by SP-NLCs, NSP-NLCs and SSP-NLCs. The percentages of ALB or CK-7 positive cells are shown in a column chart. (B) In contrast, AFP positive cells could be found after SP-HCCs, NSP-HCCs and SSP-HCCs induction. The data are summarized in a column chart. (C) By western blotting, fold differences in specific markers relative to GAPDH were analyzed in induced SP-NLCs, NSP-NLCs and SSP-NLCs. (D) Western blotting results of tumor-specific markers in induced SP-HCCs, NSP-HCCs and SSP-HCCs. Original magnification, 200× (A, B). (For a better interpretation of the colored figure, the reader is referred to the web version of the article).

Under induction conditions, each subpopulation generated distinct outgrowths. Because NLCs should differentiate into hepatocytes or biliary epithelial cells, we selected one mature hepatic marker (ALB) and one biliary marker (CK-7) to identify mature cells. Most SP-NLCs expanded into sheets of tightly packed cells that displayed typical hepatocyte morphology and were identified as ALB positive cells (66.9±5.34%). A portion of the SP-NLCs differentiated into CK-7 positive cells (24.6±2.41%) (Figure 3A). Although both NSP-NLCs and SSP-NLCs could also generate ALB positive cells, only several CK-7 positive cells could be found in induced SSP-NLCs, and no CK-7 positive cells were found in induced NSP-NLCs (Figure 3A). These data indicate that only SP-NLCs had a strong potential to differentiate into different types of mature cells. Western blotting demonstrated that although the cells generated by NSP-NLCs expressed higher ALB than the cells from SP-NLCs and SSP-NLCs, daughters of SP-NLCs expressed much higher levels of CK-7 than the daughters of SSP-NLCs and NSP-NLCs (Figure 3C). In particular, CK-7 displayed almost no expression in the daughters of NSP-NLCs (Figure 3C). These data were concordant with the IF observations.

Because HCCs should differentiate into liver tumor cells, we selected one hepatic tumor marker (AFP) and one biliary tumor marker (CK-19) to identify mature cells. After induction, the daughters of SP-HCCs, SSP-HCCs and NSP-HCCs displayed heterogeneous, differentially expressed AFP. The percentages of AFP positive cells in SP-HCCs, NSP-HCCs and SSP-HCCs were 87.8±8.65%, 65.8±5.24% and 71.5±6.13% (Figure 3B). Unfortunately, none of the three types of HCCs could generate CK-19 positive cells (Figure 3B). These data indicate that HCCs had a hepatocellular carcinoma origin. By western blotting, the expression of AFP in induced SP-HCCs was 2 times higher than that in induced NSP-HCCs and 1.5 times higher than that in induced SSP-HCCs. In contrast, CK-19 was not expressed in the daughters of SP-HCCs, SSP-HCCs and NSP-HCCs (Figure 3D). These data were concordant with the IF observations.

The daughter cells from SP-HCCs expressed much higher levels of the early hepatic marker AFP and lower levels of the late hepatic marker ALB than those of SP-NLCs (*P*<0.01). In one word, compared to NSP cells, both SP cells showed more stem-like properties (*P*<0.01).

### 5. SP-NLCs aided in treating injured livers

**Figure 4:**
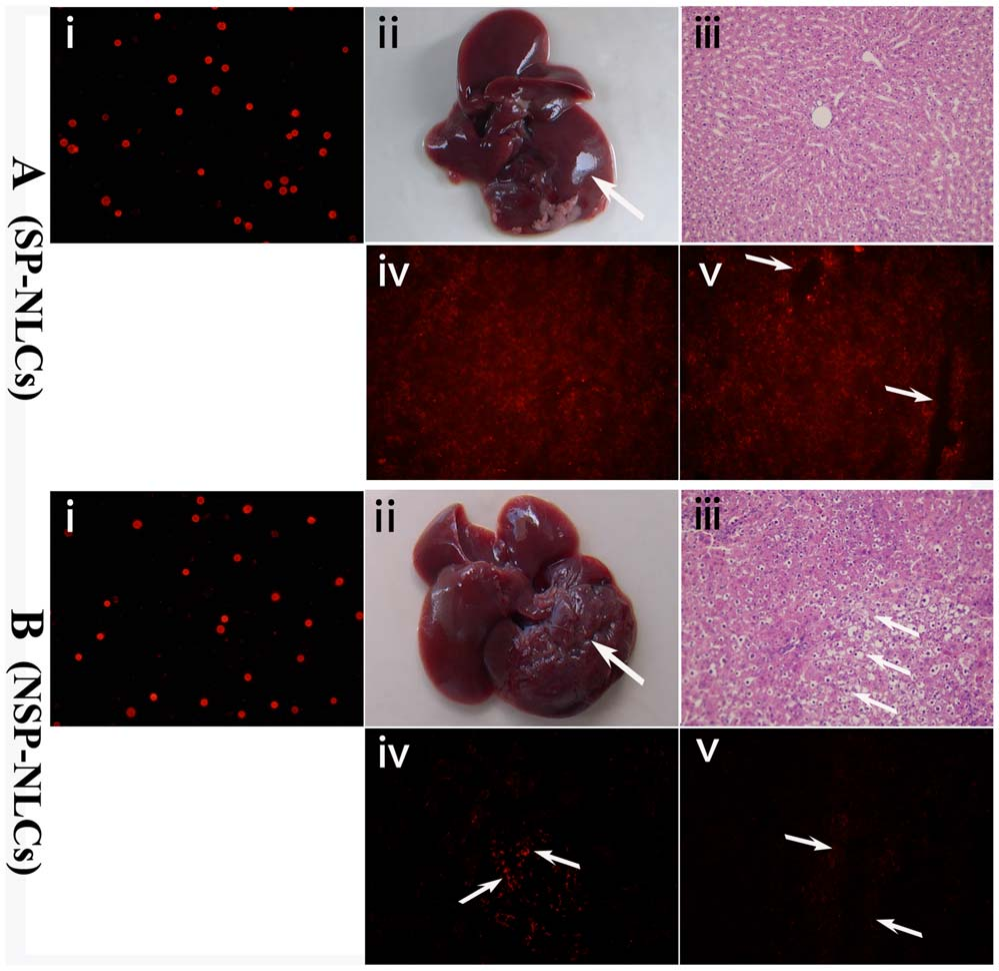
The regenerative effects of transplanted cells in acutely injured rats. The membranes of (A-i) SP-NLCs and (B-i) NSP-NLCs were successfully stained with PKH26 fluorescence. After the rats were severely damaged by CCl_4_ and a 2/3 PH, (A-ii) transplantation of SP-NLCs enhanced liver repair (shown by a smooth surface), whereas (B-ii) the livers in the NSP-NLCs injected group still exhibit a rough surface. (A-iii) The H&E staining of livers in the SP-NLCs transplanted group. (B-iii) The livers in the NSP-NLCs injected group were stained by H&E. (A-iv) After SP-NLCs transplantation, many sporadic cells labeled by red fluorescence could be observed in the liver. (B-iv) However, minor red cells could be found in NSP-NLCs transplanted liver (arrows). (A-v) Complete hepatic cord-like structure with red fluorescence could be detected in the region near the portal area of the SP-NLCs restored liver (arrows). (B-v) Around the portal area, very weak red fluorescence in NSP-NLCs repaired liver was present (arrows). Original magnification, 200× (A, B-i, iii, iv, v). (For a better interpretation of the colored figure, the reader is referred to the web version of the article).

Before transplantation, we stained the membranes of SP-NLCs (Figure 4A-i) and NSP-NLCs (Figure 4B-i) with red fluorescence using the PKH26 cell linker dye. As the dye linked the membranes of these cells, it was transferred from parent cell to the daughter cell during the process of proliferation, which occurred for up to ten generations. Before cell transplantation, the rats were severely injured by CCl_4_ and 2/3 PH. Thirty days after transplantation of the cells, the rats were sacrificed and the extent of liver repair was examined. The livers of animals receiving SP-NLCs injection had sharper edges and a smoother surface (Figure 4A-ii). In contrast, after NSP-NLCs transplantation, the livers were hardly repaired and exhibited a rough surface (Figure 4B-ii). By H&E staining, the liver tissues of the rats receiving SP-NLCs injection (Figure 4A-iii) showed fewer balloon-like morphological changes, less cell swelling and more regular cell order than those receiving an injection of the same number of NSP-NLCs (Figure 4B-iii). Under fluorescent microscopy, cells labeled by red fluorescence could be observed in SP-NLCs transplanted liver lobules (Figure 4A-iv), and branch-like red fluorescence could be detected in the region near the portal area of some lobules (Figure 4A-v). In contrast, few red cells could be observed in either the general area (Figure 4B-iv) or in the region near the portal area (Figure 4B-v) of NSP-injected liver lobules. These results demonstrate that SP-NLCs were more effectively involved in liver repair than NSP-NLCs.

Based on the grading criteria for red fluorescence in the liver sections, we analyzed 40 sections of SP-NLCs-transplanted livers and 40 sections of NSP-NLCs-injected livers. We summarized these data in Table 2. Generally speaking, most SP-NLCs restored liver sections displaying moderate or strong positive red fluorescence. In contrast, most NSP-NLCs restored liver sections reflecting negative or weak positive red fluorescence. In short, much more red fluorescence appeared in SP-NLCs-restored liver sections than in NSP-NLCs injected liver sections (*P*<0.01).

**Table 2:** The percentages of red fluorescence in liver sections.

Cell subpopulation	Sample number	Red−	Red+	Red++	Red+++
SP-NLCs	40	0±0.00	2±0.27	25±1.83	13±1.21
NSP-NLCs^a^	40	10±0.84	26±2.56	4±0.32	0±0.00

All results were viewed by three different researchers.

a NSP-NLCs *vs.* SP-NLCs, N = 40, *P*<0.01.

### 6. SP-HCCs are tumorigenic in vivo

**Figure 5:**
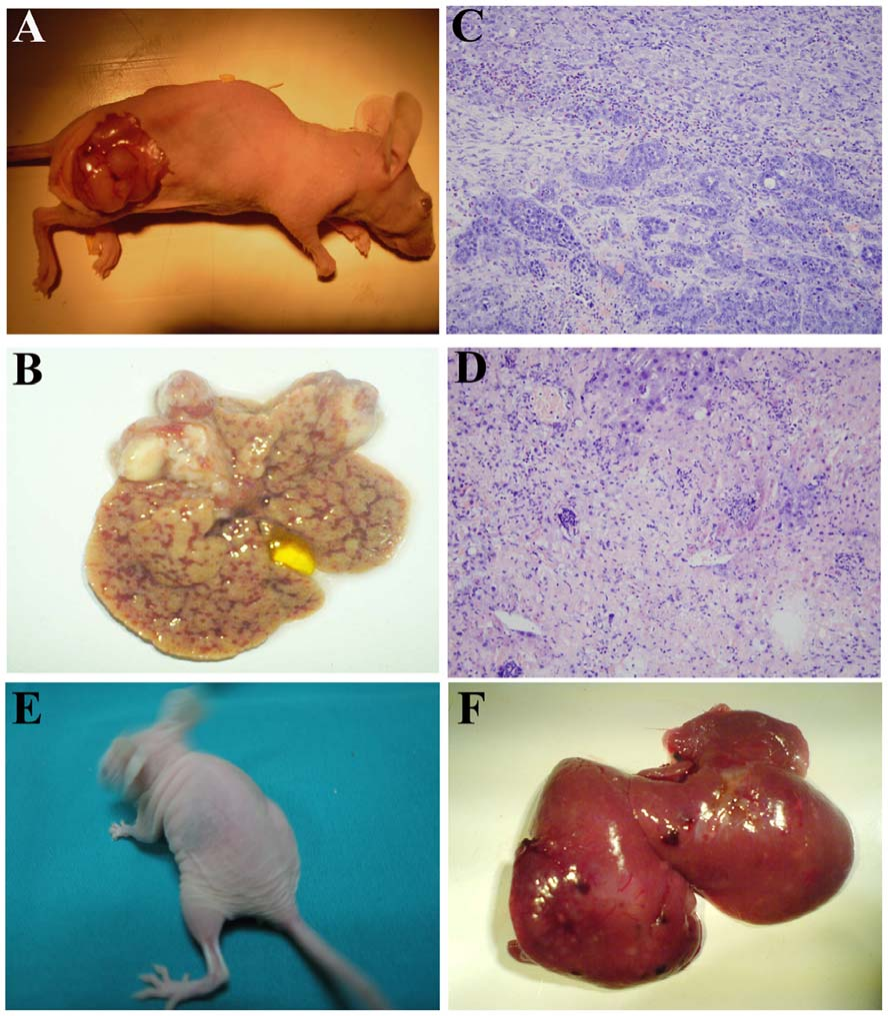
The tumor formation capacity. The smallest number SP-HCCs could generate tumors (A) not only in subcutaneous tissues (B), but also in the livers of NOD/SCID mice. Based on pathological analysis, (C) the subcutaneous tissues and (D) liver tissues underwent hepatic carcinoma genesis. In contrast, the same number NSP-HCCs could not generate tumors in (E) subcutaneous tissues or liver tissues (F) of NOD/SCID mice. Original magnification, 200× (C, D).

To test the tumorigenic ability of SP-HCCs and NSP-HCCs, various numbers of cells were injected into mice. We counted the number of tumors in each mouse, measured the size of each tumor, checked for liver metastasis, and summarized those data in Table 3. The xenograft tumors were found within nearly each mouse injected with different numbers of SP-HCCs, including those injected with as few as 1×10^4^ cells. In contrast, only more than 1×10^5^ NSP-HCCs could generate tumors. As few as 1×10^4^ SP-HCCs could initiate tumors not only in subcutaneous tissues (Figure 5A), but also in liver tissues (Figure 5B) of NOD/SCID mice. Pathological analysis indicated that the tissues from the subcutaneous regions (Figure 5C) and from the livers (Figure 5D) were all hepatic carcinoma-derived. However, the same number of NSP-HCCs (1×10^4^) could not generate tumors in subcutaneous tissues (Figure 5E) or liver tissues (Figure 5F) of NOD/SCID mice. Therefore, with the same number of cells, SP-HCCs caused more tumors and much bigger tumors than NSP-HCCs (*P*<0.01). Most importantly, liver metastasis was always present in each mouse injected with SP-HCCs. However, obvious liver metastasis could not be found in any mouse that had received the injection of NSP-HCCs (Table 3).

**Table 3:** The tumor formation of distinct typed cells in nude mice.

Cell subpopulations	Tumor incidence	Tumor diameter	Metastasis incidence
SP-HCCs (1×10^7^)	4/4	**+++**	4/4
SP-HCCs (1×10^6^)	4/4	**++**	4/4
SP-HCCs (1×10^5^)	4/4	**++**	4/4
SP-HCCs (1×10^4^)	4/4	**+**	4/4
NSP-HCCs (1×10^7^)^a^	4/4	**++**	0/4
NSP-HCCs (1×10^6^)^b^	3/4	**+**	0/4
NSP-HCCs (1×10^5^)^c^	2/4	**+**	0/4
NSP-HCCs (1×10^4^)^d^	0/4	**−**	0/4

“Tumor incidence” indicates the average incidence of tumors in each mice (4 mice in each group, 4/4 means 4 tumors in 4 injection sites). “Tumor diameter” refers to the average diameter of tumors in each group (−, no macroscopic tumor; +, <0.2 cm; ++, 0.2–0.5 cm; +++, >0.5 cm). “Metastasis incidence” means the average incidence of the liver neoplasia appeared in each group (4/4 means 4 liver neoplasias found in 4 mice). All results were independently viewed by three different researchers.

a–d NSP-HCCs *vs.* SP-HCCs (the same number), *P*<0.01.

### 7. Profile of miRNAs in SP-HCCs and SP-NLCs

**Figure 6:**
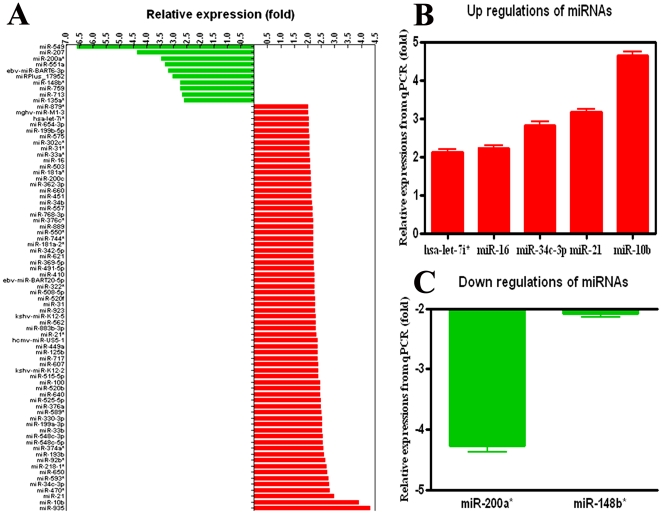
Confirmation of deregulated miRNAs by miRNA array and QRT-PCR. (A) Sixty- eight miRNAs were demonstrated to be differentially up-regulated and ten miRNAs were distinctly down-regulated using the miRNA array. (B) Five representative miRNAs displayed consistent over-expression and (C) two representative miRNAs showed concordant under-expression by QRT-PCR. The error bar indicates the SEM.

The miRNA array indicated differential expression of 78 miRNAs in SP-HCCs compared to SP-NLCs (*P*<0.01) (Figure 6A). Up-regulated miRNAs were found more frequently (87.2%; 68 of 78) than down-regulated miRNAs (12.8%; 10 of 78). The fold increase of over-expressed miRNAs varied from 2.000±0.032 to 4.319±0.312, while that of the down-regulated miRNAs was from 2.611±0.024 to 6.580±0.409. The fold change of down-regulated miRNAs was, on average, larger than that of the over-expressed miRNAs. Cluster analysis of over-expressed miRNAs (Figure S1A) and under-expressed miRNAs (Figure S1B) indicated that some deregulated miRNAs might play their roles in groups, such as up-regulated miR-10b and miR-21 and down-regulated miR-200a* and miR-148b*.

The fold change of miRNAs in SP-HCCs compared with SP-NLCs by QRT-PCR was as following (Figure 6B, C): miR-200a* (−4.275±0.094), miR-148b* (−2.087±0.050), let-7i* (2.126±0.072), miR-16 (2.227±0.076), miR-34c-5p (2.823±0.092), miR-21 (3.173±0.069) and miR-10b (4.643±0.087). The expression patterns of seven representative miRNAs detected by QRT-PCR were highly concordant with the array data.

### 8. Targets of deregulated miRNAs

The proven targets of seven validated, deregulated miRNAs are listed in Table 4. Among these targets, we detected the targets of miR-200a* (the most down-regulated miRNA) in both SP cells. In contrast to the miR-200a* expression, both targets ZEB1 and ZEB2 were expressed at much higher levels in SP-HCCs than in SP-NLCs by sQRT-PCR (Figure S2). The MiRanda miRBase uses a complementary type algorithm and the TargetScan uses a seed complementarity type algorithm. Based on these two algorithms, the top 10 putative targets for each deregulated miRNA were identified (Table S1).

**Table 4:** The proven targets of validated miRNAs.

MicroRNAs	Proven targets
**Increased expression >2-fold**	
**miR-10b**	HOXD10 [62], Tiam1 [63], PPAR-alpha [64]
**miR-21**	PTEN [65], Caspase-3 [66], PDCD4 [67]
**miR-34c-3p**	c-Met [57], c-Myc [68], E2F3 [69], BCL-2 [69]
**miR-16**	BCL2 [56], MCL1 [70], CCND1 [70], WNT3A [70], HMGA1 [71], Caprin-1 [71], Bmi-1 [72], G(1) cyclins [73]
let-7i*	TLR4 [74], E-cadherin [58], ZEB1 [58]
**Decreased expression <0.5-fold**	
miR-200a*	ZEB1 [75], ZEB2 [76], CTNNB1 [77], E-cadherin [76]
miR-148b*	None found

## Discussion

Cancer is widely accepted as a disease of stem cells because these are the only cells that persist in the tissue for a sufficient length of time to acquire the requisite number of genetic changes for neoplastic development [34]. Many researchers have demonstrated the existence of HCSCs in HCC tissues [1], [2], [3]. Accordingly, the normal liver is an excellent source of HNSCs. In this study, we successfully enriched both SP-HCCs and SP-NLCs. SP cells appeared to be enriched as stem cells, which play a pivotal role in normal development and cancer biology [16]. Thus, these cells could provide a useful tool and a readily accessible source for stem cell studies in both normal and cancerous settings [35]. In this study, both SP-NLCs and SP-HCCs were demonstrated to have a high capacity for self-renewal, high expression of stem cell markers, and multi-potency in generating different cell types. Therefore, these SP cells were stem-like cells. SP cells can thus be considered an appropriate source of stem cells [36], and comparative analysis of the characteristics that distinguish SP-HCCs and SP-NLCs would be expected to contribute to the understanding of HCC genesis. Moreover, as efficient suppressors of gene expression, miRNAs are expected to be involved in regulating the differences between SP-HCCs and SP-NLCs.

We must emphasize how we obtained the related results. After tumor was formed in F344 rats, we selected 4 rats for cell isolation. That is to say, we separately isolated HCCs from each whole HCC tissue of 4 DEN-induced rats and NLCs from each liver of 4 normal rats. The subsequent experiments were performed using HCCs or NLCs from single rat, and the results were statistically analyzed and represented as Mean ± Standard error. For example, we separately isolated SP-HCCs from each kind of 4 HCCs, SP-NLCs from each kind of 4 NLCs. The percentages of SP cells in the whole cell population were then obtained by calculating the average of the data from the 4 samples. Finally, for miRNA array, we used 4 SP-NLCs as parallel controls and 4 SP-HCCs as parallel trials.

### 1. Differences between SP-HCCs and SP-NLCs in vitro and in vivo

In this study, both SP-HCCs and SP-NLCs were demonstrated to have stem-like properties by high expression of stem cell markers. *In vitro*, because both SP cells were small and round, they could not be distinguished from each other by morphology. Although both SP-NLCs and SP-HCCs could rapidly proliferate, these cells differentiated into distinct lineages. Under induction conditions, both SP cells could be induced to differentiate into mature cells. SP-NLCs could differentiate into many ALB positive cells and a few CK-7 positive cells; in contrast, SP-HCCs could only generate AFP positive cells. These differences would result in totally different consequences *in vivo*, as described below. When the rats were severely injured by CCl_4_ and a 2/3 PH, SP-NLCs could aid in improving the injured livers both in terms of morphology and function. This rescue was through permanent implantation into the liver and differentiation into functional cells, which was concordant with previous studies [37]. In contrast, SP-HCCs had a high ability to form tumors in NOD/SCID mice, and even as few as 1×10^4^ SP-HCCs were enough to initiate HCC, which is similar to another study's findings [38]. Upon subcutaneous injection, SP-HCCs could migrate to the liver and caused neoplasias, even with only 1×10^4^ cells. However, we could not find obvious liver metastasis in any mouse that had received an injection of NSP-HCCs. These data indicate that SP-HCCs were more malignant and invasive, which may be related to their stemness. Both findings revealed that SP-NLCs were more differentiated, which was useful for the regeneration of injured livers. In contrast, SP-HCCs were more immature, suggesting that stemness may be related to invasiveness. Combining the above results, SP cells appear to serve a central role in liver regeneration and HCC genesis.

### 2. Deregulation of miRNAs in SP-HCCs and SP-NLCs

Although both SP-HCCs and SP-NLCs shared some common characteristics of stem cells, they differentiated in totally different directions that resulted in completely opposite consequences. Changes in multiple genes have been proposed to account for this process. Through *ex vivo* genetic manipulation, HNSCs can successfully generate liver carcinomas in transplanted mice [39]. During this process, mature miRNAs engage in either degradation of the target mRNA or translational repression [40]. Although the deregulated miRNAs in HCC have been detected by different researchers, the expression profile of miRNAs in HCSCs is still not understood. Thus, the analysis of miRNA expression profiles in SP-HCCs and SP-NLCs would greatly contribute to understanding HCSC genesis. For the miRNA array, we used 4 SP-NLCs as parallel controls and 4 SP-HCCs as parallel trials. Similar to the findings from carcinomas of the lung [41], ovary [42] and liver [29], our data on SP-HCCs revealed a higher frequency of miRNA over-expression than under-expression.

In this study, miR-10b, miR-21 and miR-92b were frequently over-expressed. Accordingly, these miRNAs have also been reported to have increased expression in the majority of cancer types examined [19], [41], [43], [44], [45], including HCC [46], breast [47], lung [48], colon [49] and gastric cancers [50]. In this study, miR-92b (one member of the miR-17-92 family) was highly expressed in SP-HCCs. This miRNA has been shown to control the G1/S checkpoint gene p57 and, as a result, promotes stem cell transition from G1-phase to S-phase [51]. Because the G1/S restriction is largely absent in SP cells, these cell-cycle controlling miRNAs may be responsible for enabling SP cells to rapidly move through G1 phase, enter S phase and rapidly proliferate. There are two miRNAs that are possibly related to the invasive nature of SP-HCCs. MiR-21 has been demonstrated to target PTEN [52] and results in the further modulation of HCC cell migration and invasion. This effect is believed to occur via modulation of the phosphorylation of focal adhesion kinase [52] and the expression of matrix metalloproteinases 2 and 9 [52]. Most importantly, miR-10b, the second most over-expressed miRNA in SP-HCCs, has been found to be highly expressed in metastatic breast cancer cells and has been shown to positively regulate cell migration and invasion [53]. MiR-10b inhibits the synthesis of the HOXD10 protein and permits the expression of the pro-metastatic gene product RHOC, which in turn favors cancer cell migration and invasion [53]. In short, based on previous studies, we propose that the greatly up-regulated miRNAs may contribute to the rapid proliferation, migration and invasion of SP-HCCs.

Among the moderately up-regulated miRNAs, miR-451 and miR-181a have been well studied. MiR-451, which was over-expressed in SP-HCCs, is involved in activating the expression of P-glycoprotein (P-gp), the MDR1 gene product that confers the SP phenotype [54]. In addition, miR-181a has been demonstrated to be responsible for the genesis of human liver cancer stem/progenitor cells [55]. Thus, these two miRNAs may contribute to the stem cell-like properties of SP-HCCs. However, the slightly up-regulated miR-16, miR-34c-3p and let-7i* miRNAs in this study have been demonstrated to be down-regulated in other cancer settings [56], [57], [58]. One reason for this discrepancy may result from differences in the compared objects. We compared normal stem cells to CSCs, while previous researchers have compared mature cancer tissues/cells with normal tissues/cells. In addition, the above three miRNAs may not be responsible for the differences between SP-NLCs and SP-HCCs. Moreover, the variation in the scope of miRNAs analyzed in our research was much smaller than that in other studies. Overall, we propose that these miRNAs may be marginally deregulated.

Two important miRNAs that were down-regulated in SP-HCCs, miR-200a* and miR-148b*, have been described in HCC tissues [26] and ovarian cancers [47]. Recent findings have associated miR-200a* with stem cell maintenance and suggest a connection between the epithelial-to-mesenchymal transition (EMT) and stem cell formation. A part of tumor progression can be viewed as a continuum of progressive dedifferentiation (EMT) with a cell at the endpoint that has stem cell-like properties [20]. The ZEB-miR-200 feedback loop has been demonstrated to link EMT activation and the maintenance of stemness by suppressing stemness-inhibiting miRNAs and acting as a promoter of mobile, migrating CSCs [59]. In this study, the targets of miR-200a*, ZEB1 and ZEB2 were both expressed at much higher levels in SP-HCCs than in SP-NLCs. These data indicate that the greatly down-regulated miR-200a* may promote malignant transformation of SP-NLCs and force SP-HCCs to become more metastatic.

Overall, some miRNAs are common to both NSCs and CSCs and may be required to maintain stemness [60]. However, some miRNAs that are differentially expressed between NSCs and CSCs may contribute to the distinct *in vivo* consequences caused by these two types of stem-like cells. Therefore, therapies that target the deregulation of miRNAs could be powerful tools for correcting the deregulation of CSCs [61].

### Conclusions

This study was the first to analyze the differences between SP-NLCs and SP-HCCs. Both SP cells were demonstrated to be stem-like cells. However, SP-NLCs are more regenerative, whereas SP-HCCs are more tumor-formative *in vivo*. Furthermore, given that the deregulation of miRNAs in SP-HCCs is an early event in the process of HCC genesis, this work will undoubtedly provide novel insights into the intricate relationship between miRNAs, CSCs and HCC.

### Limitations

Although we proposed that these deregulated miRNAs may contribute to the observed differences between SP-HCCs and SP-NLCs, the exact roles of these miRNAs in the process of HCSCs genesis must be verified. Several important up-regulated miRNAs and down-regulated miRNAs were found in SP-HCCs. We are planning to investigate these specific miRNAs, probe for their targets and eventually reveal the mechanisms contributing to miRNA-mediated effects on HCSC genesis.

## Supporting Information

Figure S1 **Cluster analysis of deregulated miRNAs.** (A) Cluster analysis of over-expressed miRNAs from profiling. (B) Cluster analysis of under-expressed miRNAs from profiling. Red depicts high expression levels, whereas green and black corresponded to low expression levels and non-varied signals, respectively.
(TIF)

Figure S2 The target analysis of miR-200a*. By sQRT-PCR, both target genes ZEB1 and ZEB2 were expressed at much higher levels in SP-HCCs than in SP-NLCs.
(TIF)

Table S1 **The predicted targets for deregulated miRNAs.** Based on two different algorithms, the top 10 putative targets for each deregulated miRNA were identified and summarized into a table.
(DOC)
