# Supplementary figures and images for: Differences in the Properties and Mirna Expression Profiles between Side Populations from Hepatic Cancer Cells and Normal Liver Cells
Source: PLoS One. 2011 Aug 3;6(8):e23311. doi: 10.1371/journal.pone.0023311 (PMC3149655; doi:10.1371/journal.pone.0023311)

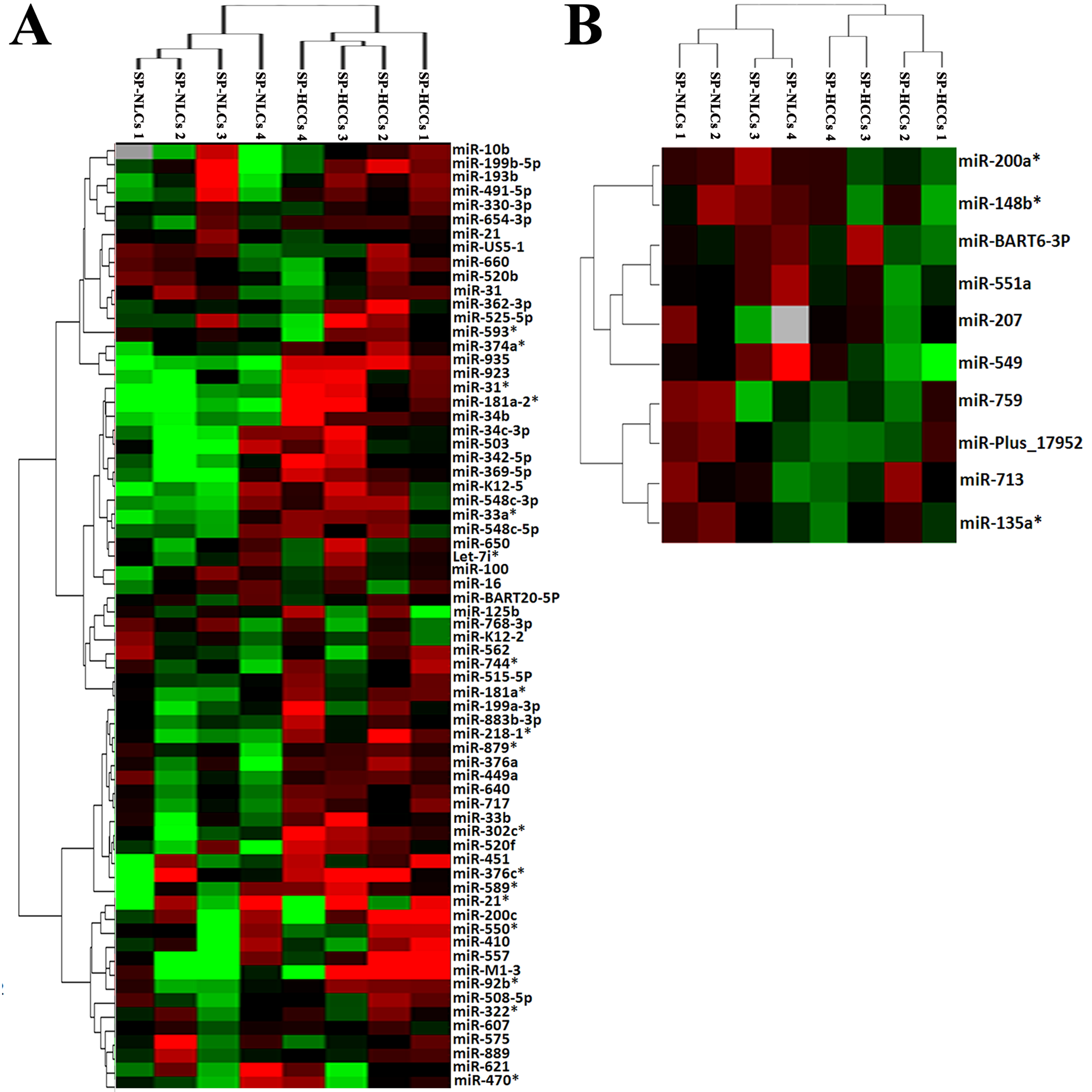

Supplement: Figure S1 — Cluster analysis of deregulated miRNAs. (A) Cluster analysis of over-expressed miRNAs from profiling. (B) Cluster analysis of under-expressed miRNAs from profiling. Red depicts high expression levels, whereas green and black corresponded to low expression levels and non-varied signals, respectively. (TIF) [file pone.0023311.s001.tif]

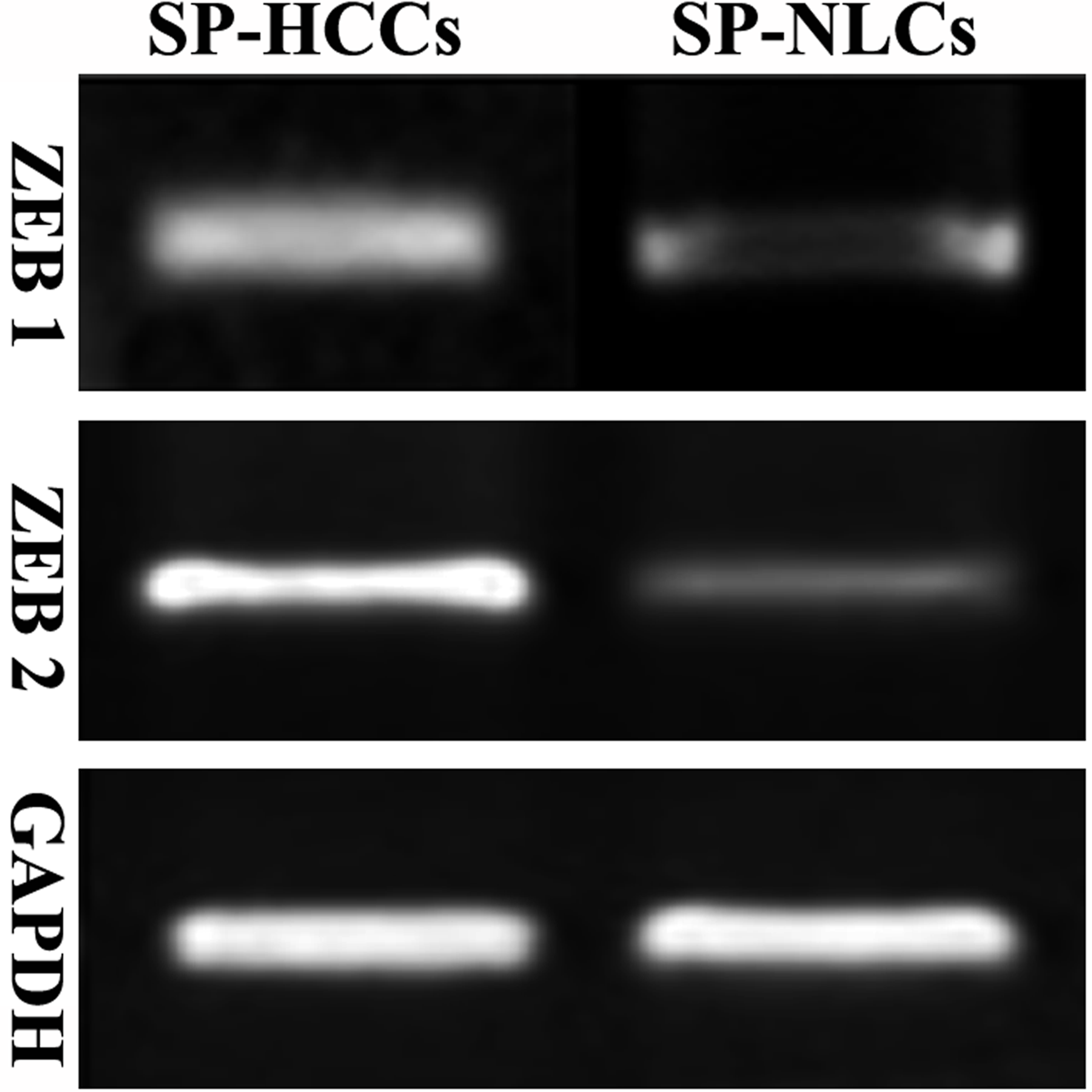

Supplement: Figure S2 — The target analysis of miR-200a*. By sQRT-PCR, both target genes ZEB1 and ZEB2 were expressed at much higher levels in SP-HCCs than in SP-NLCs. (TIF) [file pone.0023311.s002.tif]
