# Supplementary material for: Differences in the Properties and Mirna Expression Profiles between Side Populations from Hepatic Cancer Cells and Normal Liver Cells
Source: PLoS One. 2011 Aug 3;6(8):e23311. doi: 10.1371/journal.pone.0023311 (PMC3149655; doi:10.1371/journal.pone.0023311)
Supplement: Table S1 — The predicted targets for deregulated miRNAs. Based on two different algorithms, the top 10 putative targets for each deregulated miRNA were identified and summarized into a table. (DOC) [file pone.0023311.s003.doc]

| | Table S1. The predicted targets for deregulated miRNAs | | --- | | | |
| --- | --- | --- | --- |
| | **microRNA** | | --- | | **MiRBase Targets** | **Targetscan** |
| **Increased expression > 2-fold** | | |
| [**miR-10b**](http://genome-www4.stanford.edu/cgi-bin/SMD/source/sourceResult?choice=Gene&option=Name&criteria=hsa-miR-10b/mmu-miR-10b/rno-miR-10b) | **TSEN34, LSM1, HOXD10, ACPP, NCOR1** | **HCN1, TFAP2C, FIGN, ARSJ, NCOR2** |
| [**miR-21**](http://genome-www4.stanford.edu/cgi-bin/SMD/source/sourceResult?choice=Gene&option=Name&criteria=hsa-miR-21/mmu-miR-21/rno-miR-21) | **BMPR2, PTEN, ACAT1, NFIB, MSH2** | **GPR64, SCML2, PPP1R3A, SATB1, PTEN** |
| [**miR-34c-3p**](http://genome-www4.stanford.edu/cgi-bin/SMD/source/sourceResult?choice=Gene&option=Name&criteria=hsa-miR-34c-3p) | **GPX2, MTF2, AKR1C4, CXYorf2, CSPG4LYP1** | **HCN3, FAM76A, C8orf13, SATB2, PPP1R11** |
| [**miR-16**](http://genome-www4.stanford.edu/cgi-bin/SMD/source/sourceResult?choice=Gene&option=Name&criteria=hsa-miR-16/mmu-miR-16/rno-miR-16) | **GPR63, WFDC5, TAF15, SCN8A, KARS** | **FGF2,TMEM16C,PLAG1,SPRED1,KIF1B** |
| [**let-7i***](http://genome-www4.stanford.edu/cgi-bin/SMD/source/sourceResult?choice=Gene&option=Name&criteria=hsa-let-7i*/mmu-let-7i*/rno-let-7i*) | **SIX1, TMEM91, SH3TC1, CACNA1G, DNMT1** | **HMGA2, C14orf28, TRIM71, IGF2BP1, ARID3B** |
| **Decreased expression < 0.5-fold** | | |
| [**miR-200a***](http://genome-www4.stanford.edu/cgi-bin/SMD/source/sourceResult?choice=Gene&option=Name&criteria=hsa-miR-200a*/mmu-miR-200a*) | **POLR1D, RNF113B, SLC7A1, GPHA2, OLAH** | **ZEB2, KLF12, MMP16, ZFR, PRKACB** |
| [**miR-148b***](http://genome-www4.stanford.edu/cgi-bin/SMD/source/sourceResult?choice=Gene&option=Name&criteria=hsa-miR-148b*) | **Cml1, Myh1, Synj2, Cltc, Gjb6** | **PAFAH1B1, BNIP3L, SKAP2, RC3H1, FOXP1** |
| **The top 5 putative targets identified with MiRBase Targets and Targetsan were included, respectively.** | | |
